# Internet-based intervention on maternal sleep and anxiety: a randomized clinical trial

**DOI:** 10.1590/0034-7167-2025-0410

**Published:** 2026-07-31

**Authors:** Rayanne Branco dos Santos Lima, Caio Ferreira de Jesus, Lorena Pinheiro Barbosa

**Affiliations:** I Faculdade Adventista da Amazônia. Belém, Pará, Brazil; II Universidade Federal do Ceará. Fortaleza, Ceará, Brazil

**Keywords:** Sleep, Anxiety, Mothers, Internet-Based Intervention, Clinical Trial., Sueño, Ansiedad, Madres, Intervención Basada en la Internet, Ensayo Clínico.

## Abstract

**Objectives:**

to assess the effectiveness of an online intervention based on self-efficacy theory to improve sleep and reduce maternal anxiety in mothers of infants aged 10 to 24 months.

**Methods:**

a randomized clinical trial with 104 participants, allocated to intervention and control groups. The intervention group participated in two virtual meetings with guidance on sleep, relaxation, and anxiety management, in addition to motivational messages. The control group received only monitoring. Sleep quality (Pittsburgh Sleep Quality Index) and anxiety (State-Trait Anxiety Inventory) were assessed at baseline and after 15 and 30 days.

**Results:**

after 30 days, the intervention group showed significant improvement: Pittsburgh Sleep Quality Index from 10.4 (±1.8) to 4.9 (±1.2); trait-anxiety from 38.2 (±5.1) to 33.5 (±4.5); and state-anxiety from 36.4 (±4.9) to 33.8 (±4.6) (p<0.05).

**Conclusions:**

the online intervention was effective in improving maternal sleep and anxiety. Clinical trial registry: RBR-8hvdh5r.

## RESUMEN

**Objetivos:**

evaluar la eficacia de una intervención en línea basada en la teoría de la autoeficacia para mejorar el sueño y reducir la ansiedad materna en madres de bebés de 10 a 24 meses.

**Métodos:**

ensayo clínico aleatorizado con 104 participantes, asignados a los grupos de intervención y control. El grupo de intervención participó en dos reuniones virtuales con orientación sobre sueño, relajación y manejo de la ansiedad, además de mensajes motivacionales. El grupo control solo recibió monitorización. Se evaluaron la calidad del sueño (*Pittsburgh Sleep Quality Index*) y la ansiedad (Inventario de Ansiedad Estado-Rasgo) al inicio y a los 15 y 30 días.

**Resultados:**

tras 30 días, el grupo de intervención mostró una mejora significativa: *Pittsburgh Sleep Quality Index* pasó de 10,4 (±1,8) a 4,9 (±1,2); ansiedad-rasgo, de 38,2 (±5,1) a 33,5 (±4,5); y ansiedad-estado, de 36,4 (±4,9) a 33,8 (±4,6) (p<0,05).

**Conclusiones:**

la intervención en línea fue eficaz para mejorar el sueño y la ansiedad materna. Registro de ensayos clínicos: RBR-8hvdh5r

## INTRODUCTION

Sleep is a fundamental physiological need that is essential for human survival and well-being. Its importance goes beyond rest, influencing metabolic, immunological, emotional, and cognitive processes^(1)^. Sleep disorders have been associated with a number of adverse consequences, including cognitive impairment, increased risk of cardiovascular disease, and impacts on mental health such as anxiety and depression^(2,3)^. For adults, adequate sleep is essential for maintaining emotional balance and daily functioning, especially for mothers facing the challenges of their children’s first years of life^(4)^.

In a baby’s first years, mothers often experience sleep interruptions due to nighttime care demands, such as breastfeeding, diaper changes, and comforting the baby^(5)^. These interruptions are associated with poorer sleep quality and increased levels of maternal anxiety^(6)^. Studies show that maternal sleep deprivation significantly increases stress levels and diminishes positive perceptions of childcare, contributing to less effective and less satisfying parenting^(7,8)^.

The relationship between mother-infant sleep is intrinsic and bidirectional. Infants’ sleep behavior directly affects mothers’ sleep pattern, while mothers’ emotional state, including high levels of anxiety, can interfere with infant sleep regulation^(9,10)^. However, many interventions focus exclusively on infant sleep, neglecting the importance of maternal sleep care^(11,12)^. This one-dimensional approach can leave mothers vulnerable to persistent physical and mental health problems.

Furthermore, inadequate management of maternal anxiety related to sleep contributes to an exaggerated perception of threat in the face of the natural difficulties of childcare, hindering the development of self-efficacy^(13)^. Bandura’s self-efficacy theory highlights that positive emotional states are essential to increase an individual’s belief in their ability to perform actions necessary to achieve certain outcomes^(14)^. Thus, promoting interventions that strengthen maternal self-efficacy can be an effective way to improve mothers’ sleep and emotional health^(15,16)^.

In this context, educational interventions aimed at mothers, especially those mediated by technology, have shown promise. A systematic review that analyzed 44 online interventions designed to promote mental health among women during the pregnancy and postpartum period showed that these strategies were successful^(17)^.

Interventions during this period of maternal life are important and effective; however, actions aimed at mothers of older children are also necessary, especially those with children between 10 and 24 months. In this age range, babies already have greater neurological maturity to consolidate nighttime sleep, and a progressive reduction in awakenings is expected. Even so, many mothers report difficulties with infant sleep and face challenges in self-care and anxiety management. This age group, although less studied, represents a critical stage of parenthood, with demands that require new approaches to support and care^(13-18)^. There is, therefore, a gap in the literature that needs to be filled with interventions directed at this profile.

## OBJECTIVES

To assess the effectiveness of an online intervention based on self-efficacy theory in improving sleep quality and reducing anxiety scores in mothers of infants aged 10 to 24 months.

## METHODS

### Ethical aspects

This study followed the CONsolidated Standards Of Reporting Trials (CONSORT) guidelines, including the specific extension for internet-based interventions (CONSORT-EHEALTH), with the aim of ensuring transparency and quality in intervention reporting^(19)^. This study was approved by the *Universidade Federal do Ceará* Research Ethics Committee, under Opinion 5,726,949 (Certificate of Presentation for Ethical Consideration 60688822.3.0000.5054), and registered in the *Registro Brasileiro de Ensaios Clínicos* (ReBEC), under the identifier RBR-8hvdh5r. Participants signed the electronic Informed Consent Form (ICF) before starting the research. Data collection and follow-up were conducted remotely by a nurse with seven years of experience in maternal and infant sleep.

### Study design, period and place

This is a randomized controlled clinical trial with two parallel groups (intervention and control), conducted entirely remotely between March and June 2023. All activities were carried out via videoconference, using the WhatsApp^®^ and Google Meet^®^ platforms, from Fortaleza, Ceará, Brazil. Follow-up lasted 30 days, with assessments performed at baseline after 15 days and at the end of the period (30 days).

### Population or sample; inclusion and exclusion criteria

Mothers aged 18 or older whose babies were between 10 and 24 months old were invited to participate, provided they had internet access and the ability to use remote communication applications (WhatsApp^®^ and Google Meet^®^) for video calls and study participation. Those who reported a prior diagnosis of psychiatric disorders were excluded; this information was obtained through self-reporting during the completion of data collection instruments. Participants were recruited between March and June 2023 through the principal researcher’s social media. This study is part of a broader intervention on maternal and infant sleep, the results of which, concerning the babies, were published separately to facilitate understanding of the specific effects on each target group^(20)^.

Sample size was estimated based on the study’s main variable, the reduction in the number of nighttime awakenings in infants, using parameters described by Ficca *et al*. (1999)^(21)^. An initial average of 4.9 awakenings per night was considered, with a standard deviation of 2.6, and an expected minimum reduction of 3.1 awakenings after the intervention, adopting a 95% confidence level (Z₁-α/2=1.96) and a statistical power of 80% (Z₁-β=0.84). This calculation indicated a minimum need for approximately 35 mother-infant binomials per group. To reduce sample losses and increase the representativeness of the findings, all mothers who expressed interest and met the inclusion criteria during the recruitment period were included, totaling 104 eligible binomials, randomly allocated to an intervention group (IG) (n=52) and a control group (CG) (n=52). Although the initial calculation indicated a need for 70 binomials, the inclusion of a larger number was considered ethically appropriate, reinforcing the robustness of results.

### Study protocol

The study was publicized via the principal researcher’s Instagram^®^ account, which at the time had over 10,000 followers. Interested mothers were directed, via direct message or WhatsApp^®^, to the SurveyMonkey^®^ link, where they accessed the ICF and, after accepting, answered the data collection instruments: sociodemographic and clinical questionnaire; Pittsburgh Sleep Quality Index (PSQI); State-Trait Anxiety Inventory (STAI); and Brief Infant Sleep Questionnaire (BISQ). Participants had up to 48 hours to complete the questionnaires, and reminder messages were sent if there were any pending issues. If no response was received, the data was excluded from the study.

Randomization of 104 eligible participants was performed using R software (version 4.2.1) through balanced chunk randomization. Ten chunks, with ten participants each, and one chunk, with four participants, were used to maintain equivalent proportions between groups. The randomization algorithm was executed using the command “<sample()>” to generate the allocation sequence. Allocation was not blinded to the principal investigator, responsible for the intervention, but the outcomes were assessed by members blinded to the group distribution. IG participants were contacted via WhatsApp^®^ to confirm their participation and schedule the first online meeting via Google Meet^®^.

The intervention consisted of two individual online consultations conducted via videoconference. The first meeting lasted approximately 50 minutes and followed a structured script addressing adult sleep hygiene, infant sleep management, relaxation techniques, and strategies for strengthening maternal self-efficacy in the face of sleep-related anxiety. The guidance covered adult sleep hygiene practices and stimulus control, such as regular sleep and meal times, proper bed use, control of ambient lighting, room temperature and comfort, reduced use of electronics before bedtime, encouragement of aerobic physical activity, reduced consumption of stimulant beverages, and increased consumption of foods rich in tryptophan and magnesium, such as bananas and oats. Relaxation strategies were also recommended, such as therapeutic writing, deep breathing, and prayer, based on recommendations adapted from Ng and Cunnington^(22)^.

To manage maternal anxiety, healthy lifestyle habits were discussed, including sun exposure, adequate hydration, a balanced diet, physical exercise, and time dedicated to self-care. Moreover, IDATE items were addressed in a contextualized way to promote recognition and conscious management of emotional states. Throughout the meeting, visual resources such as slides and videos were used, with real-life examples of healthy maternal routines, aiming to promote vicarious experiences^(14)^. Ten days later, a second individual consultation was held, lasting approximately 30 minutes, aimed at reviewing the previous guidelines, verifying adherence to the proposed strategies, clarifying doubts, and promoting individualized adjustments, reinforcing self-confidence through verbal persuasion^(14)^.

During the 30-day follow-up period, IG participants also received short motivational messages via WhatsApp^®^. These messages followed a planned schedule: one day they addressed practices to improve baby sleep; the next day they encouraged maternal sleep care; and the third day they dealt with reducing anxiety. This three-day cycle was repeated successively throughout follow-up, ensuring continuous and varied stimulation. The content was positive and personalized, such as: *“Good morning! Remember to maintain a consistent sleep routine for your baby tonight”* or *“You’re doing a great job; try to take a moment to relax before bed tonight”*. This strategy aimed not only to reinforce maternal self-efficacy, but also to maintain the bond with participants and reduce sample losses.

CG participants did not receive guidance or counseling during follow-up. They were only contacted to complete the same assessment instruments and received reminder messages to keep a baby sleep diary, ensuring data comparability without intervention bias. At the end of the study, after data collection was completed, CG mothers were invited to participate in the same educational intervention offered to the IG, ensuring equitable access to the content.

### Outcomes

The primary outcomes of the study were maternal sleep quality and maternal anxiety. All data collection instruments were made available online and sent to participants via a link on WhatsApp^®^.

Maternal sleep quality was assessed using the PSQI, a version adapted for Brazilian Portuguese. The PSQI is a questionnaire that measures sleep quality over the past month using seven components: sleep latency; sleep duration; habitual sleep efficiency; sleep disturbances; use of sleeping medication; daytime dysfunction; and subjective sleep quality. Each component receives a score from 0 to 3, with higher values indicating greater impairment. The sum of the components generates an overall score ranging from 0 to 21 points: scores ≤5 indicate good sleep quality; scores between 6 and 9 suggest poor sleep; and scores ≥10 indicate sleep disturbance. The instrument was originally developed by Buysse *et al*. (1989)^(23)^, and translated and adapted into Brazilian Portuguese by Bertolazi *et al*. (2011)^(24)^. Brazilian studies have demonstrated adequate internal consistency (Cronbach’s α=0.71) and acceptable reliability (Intraclass Correlation Coefficient=0.65) in Brazilian adults^(25)^.

Maternal anxiety was assessed using the IDATE, translated, adapted and validated for the Brazilian adult population by Biaggio (1998)^(26)^. The instrument consists of two subscales: trait-anxiety, which assesses an individual’s general tendency to respond with anxiety to various situations; and state-anxiety, which measures the level of anxiety at the time of assessment. Each subscale contains 20 items, with responses on a Likert scale of 1 to 4 points, resulting in total scores ranging from 20 to 80. Higher values indicate greater anxiety. In the national literature, scores of 20 to 39 are considered mild/normal, 40 to 59 indicate moderate anxiety, and ≥60 suggest high or severe anxiety. In Brazil, the instrument showed satisfactory internal consistency (Cronbach’s α ≈ 0.70-0.80 in women in labor)^(27)^.

In addition to these instruments, a questionnaire specifically designed for this study was used to collect sociodemographic and clinical information from the mother and baby. This questionnaire was used to verify eligibility criteria, characterize the sample, and compare groups at baseline. The instrument was previously assessed by three pediatric nursing specialists to ensure the adequacy and clarity of its items. Maternal variables included age (in years), employment status (working outside the home: yes/no), years of education, family income, number of residents in the household, and presence of chronic or psychosomatic diseases (hypertension, diabetes, autoimmune diseases, sleep disorders, anxiety, depression, or bipolar disorder). Information was also collected on history of postpartum depression, tolerance to infant crying, and willingness to improve children’s sleep. Although the focus of this study was the mother, data on infant sleep, such as number of awakenings and total nighttime sleep time, were also obtained using the BISQ.

### Statistical analysis

Data were exported directly from the SurveyMonkey^®^ platform to Microsoft Excel^®^ spreadsheets and subjected to independent double-entry by two researchers to minimize errors. Inconsistencies were checked and corrected through cross-checking. Before analysis, the data were inspected for outliers using boxplots and z-scores. No values were excluded as they did not have a significant impact on the distribution. Continuous variables were kept in numerical format, and categorical variables were coded with binary or ordinal values, depending on the type of analysis required.

The normality of distributions was verified using the Shapiro-Wilk test. Descriptive statistics (means, standard deviations, absolute frequencies, and relative frequencies) were used to characterize the sample. Comparisons between the IG and CG at baseline and at different assessment times were performed using t-test for independent samples for continuous variables and chi-square test for categorical variables. To analyze differences within each group over time (preand post-intervention), paired t-test was used. The association among continuous variables was assessed using Pearson’s correlation. The significance level adopted was 5% (p < 0.05). All analyses were performed using R software (version 4.2.1).

## RESULTS

Following the dissemination of the study on social media, 120 women expressed interest and completed the data collection instruments between March and June 2023. Of these, 16 were excluded for not meeting inclusion criteria related to the characteristics of their children. Thus, 104 mother-infant pairs were included and randomized, with 52 allocated to the IG and 52 to the CG. Initial assessment (baseline) was performed before the start of the intervention. IG participants were reassessed 15 and 30 days after the intervention, while CG participants were reassessed 15 and 30 days after confirmation of participation.

**Figure 1:**
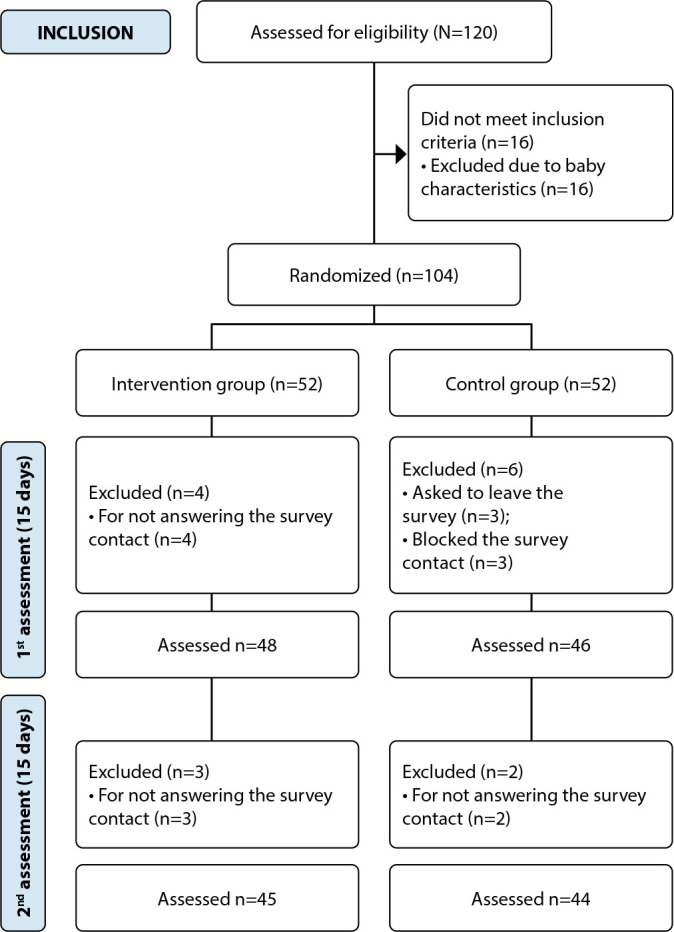
Flowchart for recruiting and allocating study participants, Fortaleza, Ceará, Brazil, 2023

Figure 1 presents the flowchart for recruitment and follow-up of participants in each phase of the study. Outcome assessments occurred at two time points: 15 and 30 days after the intervention for the IG; and after confirmation of participation for the CG.

At baseline, homogeneity was observed between the IG and CG regarding mothers’ sociodemographic variables (p>0.05), as shown in Table 1. Considering mothers’ sociodemographic characteristics in both groups, the mean age was 31.2 (±3.6) years; 59.6% had an income of three or more minimum wages; 75% had 15 or more years of education; 57.7% did not work outside the home; and 75% resided in northeastern Brazil.

**Table 1:** Mothers’ sociodemographic and clinical characteristics according to the intervention and control groups at baseline, Fortaleza, Ceará, Brazil, 2023 (N = 104)

Variables	Intervention group (n=52)		Control group (n=52)		*p* value
	Mean	Standard deviation	Mean	Standard deviation	
Mother’s age (years)	31.6	3.7	30.8	3.5	0.261^1^
Income (wage of R$1,302.00)^**^	3255.00	835	3330.00	791	0.639^1^
Mother’s education level (years)	14.9	1.89	15	1.74	0.872^1^
	n	(%)	n	(%)	
Works outside the home					
Yes	23	52.3	21	47.7	0.843^2^
No	29	48.3	31	51.7	
Region of the country					
North	4	7.7	1	1.9	0.362^3^
Northeast	37	71.2	41	78.8	
Central-West	2	3.8	-	-	
Southeast	8	14.4	8	15.4	
South	1	1.9	2	3.8	

1 t-test for independent samples;

2 chi-square for homogeneity;

3 Pearson’s chi-square test with Bonferroni correction;

** The minimum wage in Brazil at the time of data collection was R$ 1,302.00 (approximately US$ 234, EUR 221 or GBP 182).

**Figure 2:**
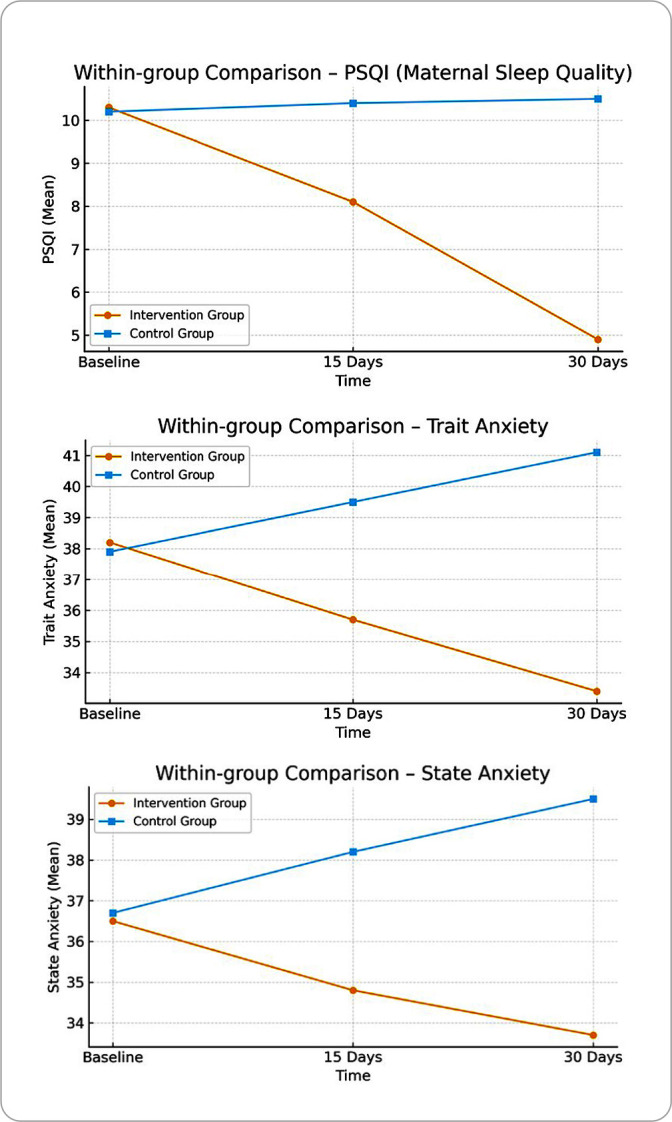
Comparison of mean sleep quality (Pittsburgh Sleep Quality Index) and anxiety scores (State-Trait Anxiety Inventory) in intervention and control groups over time, Fortaleza, Ceará, Brazil, 2023

Intragroup analysis, presented in Figure 2, showed a progressive and significant improvement in sleep quality and maternal anxiety in the IG over the 30 days. The PSQI showed a sharp decline between baseline and day 30 (p<0.001), while in the CG, scores remained stable. For trait-anxiety, a significant reduction was observed in the IG (p<0.001), contrasting with the progressive increase in the CG (p=0.012). State-anxiety followed a similar pattern, with improvement in the IG (p=0.015) and worsening in the CG (p=0.011).

Table 2 presents the comparison between the IG and CG based on the total scores of the PSQI and IDATE. The classification according to the instruments’ cut-off points is also presented. At baseline, there were no significant differences between groups in sleep scores (PSQI) and anxiety (trait and state), with both groups presenting sleep disorders and moderate anxiety. After 15 days, a significant improvement in sleep quality was observed in the IG (p<0.001), with a mean score indicating poor sleep and a reduction in state-anxiety (p=0.015). At 30 days, the IG reached a score compatible with good sleep quality (p<0.001), in addition to presenting lower levels of trait-anxiety (p=0.009) and state-anxiety (p=0.007) compared to the CG, although the overall anxiety classification remained moderate.

**Table 2:** Intergroup comparison based on Pittsburgh Sleep Quality Index and State-Trait Anxiety Inventory scores at baseline with 15 and 30 days after the intervention, Fortaleza, Ceará, Brazil, 2023

	Total PSQI	Classification	Trait-anxiety	Classification	State-anxiety	Classification
**Time/group**	**Mean/SD**		**Mean/SD**		**Mean/SD**	
**Before intervention**						
Intervention group n=52	10.8±4.2	Presence of sleep disorder	46.5±6.5	Moderate anxiety	43.6±6.3	Moderate anxiety
Comparison group n=52	11.7±3.9	Presence of sleep disorder	46.9±6.3	Moderate anxiety	44.2±6.4	Moderate anxiety
*p* value	0.292		0.774		0.610	
**15 days after intervention**						
Intervention group n=48	7.2±3.0	Poor sleep	45.6±6.7	Moderate anxiety	42.9±4.3	Moderate anxiety
Comparison group n=46	11.1±3.7	Presence of sleep disorder	47.7±6.6	Moderate anxiety	45.8±5.7	Moderate anxiety
*p* value	**<0.001**		0.186		**0.015**	
**30 days after intervention**						
Intervention group n=45	4.8±2.0	Good quality	44.9±6.3	Moderate anxiety	42.8±3.1	Moderate anxiety
Comparison group n=44	11.3±2.0	Presence of sleep disorder	48.5±6.4	Moderate anxiety	48.8±5.1	Moderate anxiety
*p* value	**<0.001**		**0.009**		**0.007**	

* *t-test for independent samples; DP - standard deviation; PSQI - Pittsburgh Sleep Quality Index.*

Table 3 shows correlations between infant sleep variables and maternal outcomes in the IG. A negative correlation was observed between the baby’s nighttime sleep time and maternal PSQI scores at 15 and 30 days, indicating that the longer the baby’s sleep, the better the mother’s sleep quality. There was also a positive correlation between the number of baby awakenings and maternal PSQI scores, suggesting poorer sleep quality with more interruptions. Furthermore, maternal PSQI correlated positively with trait-anxiety scores at both assessed time points. No significant correlations were found with state-anxiety.

**Table 3:** Correlation between baby’s nighttime sleep duration, sleep awakenings, mothers’ sleep quality, and anxiety state-trait in the intervention group, Fortaleza, Ceará, Brazil, 2023

		Mothers’ sleep quality	Mothers’ trait-anxiety	Mothers’ state-anxiety
**15 days after intervention**				
Baby’s nighttime sleep time	r^+^	-0.413	-0.252	-0.122
	p^*^	**0.004**	0.84	0.414
Baby’s night awakenings	r^+^	0.338	0.239	0.192
	p^*^	**0.019**	0.102	0.195
Mothers’ sleep quality	r^+^	-	0.430	0.135
	p^*^		**0.003**	0.370
**30 days after intervention**				
Baby’s nighttime sleep time	r^+^	-0.422	-0.009	0.029
	p^*^	**0.004**	0.957	0.949
Baby’s night awakenings	r^+^	0.434	0.082	-0.37
	p^*^	**0.004**	0.599	0.812
Mothers’ sleep quality	r^+^	-	0.492	-0.68
	p^*^		**0.001**	0.665

* *Pearson’s correlation; + Pearson’s correlation coefficient.*

## DISCUSSION

This study stands out for its innovative approach in integrating online educational strategies to improve sleep quality and reduce maternal anxiety. The implementation of practical guidelines, such as sleep hygiene, relaxation techniques, and specific interventions for maternal sleep and anxiety, demonstrates the feasibility of remote interventions for mothers. This model not only addresses immediate sleep-related issues but also broadens horizons by suggesting that other topics, such as mental health and general well-being, could be addressed in a similar format.

The choice of a nurse specializing in sleep was one of the key differentiators of the research. This continuous support helped IG mothers implement behavioral changes more effectively, resulting in a significant reduction in PSQI and trait-anxiety scores. The mothers received detailed and personalized guidance, which strengthened adherence to the proposed practices and contributed to consistent results throughout the study. The importance of specialized monitoring is widely corroborated by the literature, which highlights that interventions conducted by qualified nurses increase the effectiveness of sleep education programs^(28,29)^.

At the beginning of the study, IG and CG mothers presented PSQI scores > 10, indicating a sleep disorder. After 15 days, IG mothers had already reduced their scores to “poor sleep”, and at 30 days, they achieved “good quality” (PSQI < 5). In contrast, CG mothers maintained high scores, reinforcing the positive impact of the intervention. Furthermore, IG mothers showed consistent reductions in trait-anxiety, although state-anxiety remained stable, possibly due to external influences not directly related to the variables measured in the study.

The online meetings allowed for dynamic and accessible learning, expanding the program’s reach to mothers in different geographic contexts across Brazil. This approach is especially relevant in an increasingly digital world, where geographical barriers can be overcome through the use of technology. Previous studies have already shown that digital platforms are effective in promoting maternal and child health and well-being, especially when combined with professional guidance^(30,31)^.

On social media, healthcare professionals have demonstrated the ability to influence and engage people from diverse sociocultural backgrounds. This communication and relationship-building skill may have contributed to participants’ high engagement and low sample loss rate throughout the follow-up of this study. Similar health promotion strategies can be adapted to the context of Basic Health Units, where nurses and other professionals do not necessarily need to use their personal social networks, but can mobilize their active presence, therapeutic bond, and community leadership to implement effective educational actions. This approach can include topics that are still little explored in primary care, such as maternal and infant sleep, expanding comprehensive care and strengthening mental health and family well-being promotion.

Additionally, the relationship between infant and maternal sleep was confirmed by the observed correlations. Infants’ nighttime sleep duration showed a negative correlation with mothers’ PSQI (ρ=-0.413 at 15 days; ρ=-0.422 at 30 days), while nighttime awakenings showed a positive correlation (ρ=0.338 and ρ=0.434, respectively). These findings support studies that highlight the interdependence between infants’ and mothers’ sleep quality, as reported by Cai *et al*.^(9)^ and Sánchez-Garcia *et al*.^(32)^.

Another relevant contribution of this study was the strengthening of maternal self-efficacy. Although this construct was not directly measured, the entire educational intervention was based on its principles, with the aim of promoting mothers’ confidence in their ability to cope with the challenges related to improving sleep quality and reducing anxiety. This focus on self-efficacy is a critical factor for the success of behavioral interventions, as also demonstrated by Schnatschmidt *et al*.^(16)^ and Carroll *et al*.^(13)^.

Future studies should explore the long-term sustainability of these results, investigating whether improvements in sleep quality and anxiety reduction are maintained after the intervention. Furthermore, it would be valuable to apply this approach to more diverse populations, considering cultural and socioeconomic variables that may influence the outcomes. Integrating this intervention into public health programs also represents a promising opportunity to broaden its impact and benefit a larger number of families.

It is important to highlight the relevance of integrating strategies targeting maternal sleep and anxiety, as well as infant sleep, into existing maternal and child health policies, such as the Stork Network and childcare programs. The inclusion of specific actions in these protocols, such as education on sleep hygiene, early identification of signs of emotional distress, and motivational support, can contribute to preventing complications arising from sleep deprivation, whose relationship with adverse outcomes in maternal and infant populations has already been widely demonstrated^(7,8)^. This study reaffirms that online educational interventions, conducted by specialized professionals, can promote significant changes in mothers and infants’ quality of life. By addressing complex issues such as sleep and anxiety in an accessible and evidence-based manner, this research establishes a model that can be replicated and expanded to other areas of maternal and child health.

### Study limitations

This study has limitations that should be considered. Data collection was carried out using self-reported instruments, which may generate memory bias and social desirability biases, despite the use of validated scales. The registration of the research on the ReBEC platform occurred after the start of data collection due to technical instabilities, although the protocol was initiated in November 2022 and followed as approved by a Research Ethics Committee. The sample, although statistically adequate, was composed mainly of women from the Brazilian northeast with a similar socioeconomic profile, which limits the generalizability of the findings. Furthermore, the 30-day follow-up period prevented the assessment of the long-term sustainability of the effects, suggesting the need for future studies with greater sample diversity and prolonged follow-up.

### Contributions to nursing, health, or public policy

This study offers a relevant contribution to nursing by demonstrating that online educational interventions can be effective in promoting sleep health and reducing maternal anxiety. The results broaden the possibilities for nurses to act, both by including the topic of sleep and maternal mental health in their field of care and by exploring new ways to promote health through accessible technologies.

It also helps expand the themes addressed in existing public policies for maternal and child health in Brazil.

## CONCLUSIONS

The results of this study confirm the effectiveness of an online educational intervention conducted by a specialized professional in improving sleep quality and reducing maternal anxiety. The self-efficacy-focused approach, combined with technical and emotional support, proved to be a valuable tool for addressing the challenges of maternal sleep. In addition to promoting immediate benefits, this intervention offers a replicable and scalable model for other maternal and child health contexts, highlighting the importance of including these strategies in public policies aimed at family well-being. Future studies should explore the sustainability of the results and the expansion of this approach to more diverse populations.

## Data Availability

The research data are available only upon request.
