# Supplementary material for: Internet-based intervention on maternal sleep and anxiety: a randomized clinical trial
Source: Rev Bras Enferm. 2026 Jul 31;79:e20250410. doi: 10.1590/0034-7167-2025-0410 (PMC13435862; doi:10.1590/0034-7167-2025-0410)
Supplement: Translation

# Translation: Intervenção baseada em internet sobre sono e ansiedade materna: ensaio clínico randomizado

**DOI:** 10.1590/0034-7167-2025-0410pt

**Authors:** Rayanne Branco dos Santos Lima, Caio Ferreira de Jesus, Lorena Pinheiro Barbosa

**Affiliations:** I Faculdade Adventista da Amazônia. Belém, Pará, Brasil; II Universidade Federal do Ceará. Fortaleza, Ceará, Brasil

**Keywords:** Sono, Ansiedade, Mães, Intervenção Baseada em Internet, Ensaio Clínico.

## Abstract

**Objetivos:**

avaliar a eficácia de intervenção *online* baseada na teoria da autoeficácia para melhorar o sono e reduzir ansiedade materna em mães de bebês de 10 a 24 meses.

**Métodos:**

ensaio clínico randomizado com 104 participantes, alocadas em grupo intervenção e controle. O grupo intervenção participou de dois encontros virtuais com orientações sobre sono, relaxamento e manejo da ansiedade, além de mensagens motivacionais. O grupo controle recebeu apenas acompanhamento. A qualidade do sono (*Pittsburgh Sleep Quality Index*) e ansiedade (Inventário de Ansiedade Traço-Estado) foram avaliadas na linha de base após 15 e 30 dias.

**Resultados:**

após 30 dias, o grupo intervenção apresentou melhora significativa: *Pittsburgh Sleep Quality Index* de 10,4 (±1,8) para 4,9 (±1,2); ansiedade-traço de 38,2 (±5,1) para 33,5 (±4,5); e ansiedade-estado de 36,4 (±4,9) para 33,8 (±4,6) (p<0,05).

**Conclusões:**

a intervenção *online* foi eficaz para melhorar o sono e ansiedade materna. Registro do ensaio clínico: RBR-8hvdh5r.

## INTRODUÇÃO

O sono é uma necessidade fisiológica fundamental, essencial para a sobrevivência humana e bem-estar. Sua relevância vai além do descanso, influenciando processos metabólicos, imunológicos, emocionais e cognitivos^(1)^. Distúrbios no sono têm sido associados a uma série de consequências adversas, incluindo prejuízos cognitivos, aumento de risco para doenças cardiovasculares e impactos na saúde mental, como ansiedade e depressão^(2,3)^. Para adultos, o sono adequado é indispensável para sustentar o equilíbrio emocional e a funcionalidade diária, especialmente para mães que enfrentam os desafios dos primeiros anos de vida de seus filhos^(4)^.

Nos primeiros anos do bebê, as mães frequentemente experimentam interrupções no sono devido às demandas de cuidado noturno, como amamentação, trocas de fraldas e consolo do bebê^(5)^. Essas interrupções estão associadas a uma pior qualidade do sono e a um aumento dos níveis de ansiedade materna^(6)^. Estudos demonstram que a privação de sono materna eleva significativamente os níveis de estresse e diminui a percepção positiva do cuidado infantil, contribuindo para uma parentalidade menos eficaz e menos satisfatória^(7,8)^.

A relação entre o sono da díade mãe-bebê é intrínseca e bidirecional. O comportamento de sono do bebê afeta diretamente o padrão de sono da mãe, enquanto o estado emocional materno, incluindo altos níveis de ansiedade, pode interferir na regulação do sono infantil^(9,10)^. Contudo, muitas intervenções focam exclusivamente no sono do bebê, negligenciando a importância do cuidado com o sono materno^(11,12)^. Essa abordagem unidimensional pode deixar as mães vulneráveis a problemas persistentes de saúde física e mental.

Além disso, o manejo inadequado da ansiedade materna relacionada ao sono contribui para uma percepção exagerada de ameaça diante das dificuldades naturais do cuidado infantil, prejudicando o desenvolvimento da autoeficácia^(13)^. A teoria da autoeficácia de Bandura destaca que estados emocionais positivos são essenciais para aumentar a crença de um indivíduo em sua capacidade de executar ações necessárias para alcançar determinados resultados^(14)^. Assim, promover intervenções que fortaleçam a autoeficácia materna pode ser um caminho eficaz para melhorar tanto o sono quanto a saúde emocional das mães^(15,16)^.

Nesse contexto, intervenções educativas direcionadas às mães, especialmente àquelas mediadas por tecnologia, têm se mostrado promissoras. Estudo de revisão sistemática que analisou 44 intervenções *online* destinadas a promover saúde mental a mulheres no período gravídico-puerperal evidenciou que essas estratégias obtiveram sucesso^(17)^.

Intervenções nesse período da vida materna são importantes e eficazes; contudo, também se fazem necessárias ações voltadas para mães de crianças maiores, especialmente aquelas com filhos entre 10 e 24 meses. Nessa faixa etária, os bebês já apresentam maior maturidade neurológica para consolidar o sono noturno, sendo esperada uma redução progressiva dos despertares. Ainda assim, muitas mães relatam dificuldades com o sono infantil e enfrentam desafios no autocuidado e na gestão da ansiedade. Esse grupo etário, embora menos estudado, representa uma etapa crítica da parentalidade, com demandas que exigem novas abordagens de apoio e cuidado^(13-18)^. Há, portanto, uma lacuna na literatura que precisa ser preenchida com intervenções direcionadas a esse perfil.

## OBJETIVOS

Avaliar a eficácia de intervenção *online* baseada na teoria da autoeficácia para melhorar a qualidade de sono e diminuir os escores de ansiedade em mães de bebês de 10 a 24 meses.

## MÉTODOS

### Aspectos éticos

O presente estudo seguiu integralmente as diretrizes dos *CONsolidated Standards Of Reporting Trials* (CONSORT), incluindo a extensão específica para intervenções baseadas na internet (CONSORT-EHEALTH), com o objetivo de garantir transparência e qualidade no relato da intervenção^(19)^. Foi aprovado pelo Comitê de Ética em Pesquisa da Universidade Federal do Ceará, sob o Parecer nº 5.726.949 (Certificado de Apresentação para Apreciação Ética nº 60688822.3.0000.5054), e registrado no Registro Brasileiro de Ensaios Clínicos (ReBEC), sob o identificador RBR-8hvdh5r. As participantes assinaram o Termo de Consentimento Livre e Esclarecido (TCLE) eletrônico antes de iniciarem a pesquisa. A coleta de dados e o acompanhamento foram realizados de forma remota por uma enfermeira com sete anos de experiência em sono materno-infantil.

### Desenho, período e local do estudo

Trata-se de ensaio clínico randomizado controlado, com dois grupos paralelos (intervenção e controle), conduzido integralmente de forma remota entre março e junho de 2023. Todas as atividades foram realizadas por videoconferência, com o apoio das plataformas *WhatsApp* ^®^ e *Google Meet* ^®^, a partir de Fortaleza, Ceará, Brasil. O seguimento teve duração de 30 dias, com avaliações realizadas na linha de base após 15 dias e ao final do período (30 dias).

### População ou amostra; critérios de inclusão e exclusão

Foram convidadas a participar mães com idade igual ou superior a 18 anos, cujos bebês tinham entre 10 e 24 meses, com acesso à internet e capacidade de utilizar aplicativos de comunicação remota (*WhatsApp* ^®^ e *Google Meet* ^®^) para chamadas de vídeo e acompanhamento do estudo. Foram excluídas aquelas que relataram diagnóstico prévio de transtornos psiquiátricos, informação obtida por autodeclaração no momento do preenchimento dos instrumentos de coleta. As participantes foram recrutadas entre março e junho de 2023 por meio das redes sociais da pesquisadora principal. Este estudo integra uma intervenção mais ampla sobre sono materno-infantil, cujos resultados referentes aos bebês foram divulgados separadamente, com o objetivo de facilitar a compreensão dos efeitos específicos em cada público-alvo^(20)^.

O tamanho da amostra foi estimado com base na variável principal do estudo, redução do número de despertares noturnos dos bebês, utilizando parâmetros descritos por Ficca *et al*.^(21)^. Consideraram-se uma média inicial de 4,9 despertares por noite, com desvio padrão de 2,6, e expectativa de redução mínima de 3,1 despertares após a intervenção, adotando nível de confiança de 95% (Z₁-α/2=1,96) e poder estatístico de 80% (Z₁-β=0,84). Esse cálculo indicou a necessidade mínima de aproximadamente 35 díades mãe-bebê por grupo. Para reduzir perdas amostrais e ampliar a representatividade dos achados, foram incluídas todas as mães que manifestaram interesse e atenderam aos critérios de inclusão durante o período de recrutamento, totalizando 104 díades elegíveis, alocadas aleatoriamente em grupo intervenção (GI) (n=52) e grupo controle (GC) (n=52). Embora o cálculo inicial indicasse a necessidade de 70 díades, a inclusão de um número maior foi considerada eticamente adequada, reforçando a robustez dos resultados.

### Protocolo do estudo

A divulgação do estudo ocorreu via *Instagram* ^®^ da pesquisadora principal, que à época contava com mais de 10 mil seguidores. Mães interessadas foram direcionadas, por mensagem direta ou *WhatsApp* ^®^, ao *link* do *SurveyMonkey* ^®^, onde acessaram o TCLE e, após o aceite, responderam aos instrumentos de coleta: questionário sociodemográfico e clínico; *Pittsburgh Sleep Quality Index* (PSQI); Inventário de Ansiedade Traço-Estado (IDATE); e *Brief Infant Sleep Questionnaire* (BISQ). As participantes tinham até 48 horas para finalizar os questionários, sendo enviadas mensagens de lembrete em caso de pendência. Persistindo a ausência de resposta, os dados eram excluídos da pesquisa.

A randomização das 104 participantes elegíveis foi realizada no *software* R (versão 4.2.1) por meio de randomização em blocos balanceados, sendo utilizados dez blocos, com dez participantes cada, e um bloco, com quatro participantes, a fim de manter proporções equivalentes entre os grupos. O algoritmo de randomização foi executado com o comando “<*sample*()>” para gerar a sequência de alocação. A alocação não foi cega para a pesquisadora principal, responsável pela intervenção, mas os desfechos foram avaliados por membros cegos quanto à distribuição dos grupos. As participantes do GI foram contatadas via *WhatsApp* ^®^ para confirmar participação e agendar o primeiro encontro *online* pelo *Google Meet* ^®^.

A intervenção foi composta por duas consultas *online* individuais, realizadas por videoconferência. O primeiro encontro teve duração aproximada de 50 minutos, e seguiu um roteiro estruturado abordando higiene do sono adulto, manejo do sono infantil, técnicas de relaxamento e estratégias para fortalecimento da autoeficácia materna diante da ansiedade relacionada ao sono. As orientações abordaram práticas de higiene do sono adulto e controle de estímulos, como regularidade nos horários de sono e alimentação, uso correto da cama, controle de luzes no ambiente, temperatura e conforto do quarto, redução do uso de eletrônicos antes do horário de dormir, incentivo à atividade física aeróbica, redução do consumo de bebidas estimulantes, e aumento do consumo de alimentos ricos em triptofano e magnésio, tais como banana e aveia. Recomendaram-se ainda estratégias de relaxamento, como escrita terapêutica, respiração profunda e orações, conforme recomendações adaptadas de Ng e Cunnington^(22)^.

Para o manejo da ansiedade materna, foram discutidos hábitos saudáveis de vida, incluindo exposição solar, hidratação adequada, alimentação equilibrada, exercício físico e tempo dedicado ao autocuidado. Além disso, os itens do IDATE foram abordados de forma contextualizada para promover reconhecimento e manejo consciente dos estados emocionais. Durante todo o encontro, foram utilizados recursos visuais, como *slides* e vídeos, com exemplos reais de rotinas maternas saudáveis, com o objetivo de promover experiências vicárias^(14)^. Dez dias depois, foi realizada uma segunda consulta individual, com cerca de 30 minutos, destinada a revisar as orientações anteriores, verificar a adesão às estratégias propostas, esclarecer dúvidas e promover ajustes individualizados, reforçando a autoconfiança por meio de persuasão verbal^(14)^.

Durante os 30 dias de acompanhamento, as participantes do GI também receberam mensagens motivacionais curtas via *WhatsApp* ^®^. Essas mensagens seguiram um cronograma planejado: em um dia, abordavam práticas para melhorar o sono do bebê; no dia seguinte, motivavam o cuidado com o sono materno; e no terceiro dia, tratavam da redução da ansiedade. Esse ciclo de três dias se repetiu sucessivamente durante todo o acompanhamento, garantindo estímulo contínuo e variado. Os conteúdos eram positivos e personalizados, por exemplo: *“Bom dia! Lembre-se de manter uma rotina de sono consistente para o seu bebê esta noite”* ou *“Você está fazendo um ótimo trabalho; tente reservar um momento para relaxar antes de dormir hoje”*. Essa estratégia visou não apenas reforçar a autoeficácia materna, mas também manter o vínculo com as participantes e reduzir perdas amostrais.

As participantes do GC não receberam orientação ou aconselhamento durante o acompanhamento. Elas eram contatadas apenas para responder aos mesmos instrumentos de avaliação e recebiam mensagens de lembrete para manter um diário de sono do bebê, garantindo comparabilidade dos dados sem viés de intervenção. Ao final do estudo, após a conclusão da coleta de dados, as mães do GC foram convidadas a participar da mesma intervenção educativa oferecida ao GI, assegurando acesso equitativo ao conteúdo.

### Desfechos

Os desfechos primários do estudo foram a qualidade do sono materno e a ansiedade materna. Todos os instrumentos de coleta foram disponibilizados em formato *online* e enviados às participantes por *link* via *WhatsApp* ^®^.

A qualidade do sono materno foi avaliada pelo PSQI, versão adaptada para o português do Brasil. O PSQI é um questionário que mensura a qualidade do sono no último mês por meio de sete componentes: latência do sono; duração do sono; eficiência habitual; interrupções noturnas; uso de medicamentos para dormir; disfunção diurna; e percepção subjetiva da qualidade do sono. Cada componente recebe pontuação de 0 a 3, sendo que valores mais altos indicam maior comprometimento. A soma dos componentes gera um escore global que varia de 0 a 21 pontos: escores ≤5 indicam boa qualidade do sono; escores entre 6 e 9 sugerem sono ruim; e escores ≥10 indicam distúrbio do sono. O instrumento foi originalmente desenvolvido por Buysse *et al*. (1989)^(23)^, sendo traduzido e adaptado para o português brasileiro por Bertolazi *et al*. (2011)^(24)^. Estudos nacionais demonstraram consistência interna adequada (α de Cronbach=0,71) e confiabilidade aceitável (Coeficiente de Correlação Intraclasse=0,65) em adultos brasileiros^(25)^.

A ansiedade materna foi avaliada pelo IDATE, traduzido, adaptado e validado para a população adulta brasileira por Biaggio (1998)^(26)^. O instrumento é composto por duas subescalas: ansiedade-traço, que avalia a tendência geral do indivíduo a responder com ansiedade a diversas situações; e ansiedade-estado, que mensura o nível de ansiedade no momento da avaliação. Cada subescala contém 20 itens, com respostas em escala Likert de 1 a 4 pontos, resultando em escores totais que variam de 20 a 80. Valores mais altos indicam maior ansiedade. Na literatura nacional, escores de 20 a 39 são considerados leves/normais, de 40 a 59, indicam ansiedade moderada, e ≥60, sugerem ansiedade elevada ou severa. No Brasil, o instrumento apresentou consistência interna satisfatória (α de Cronbach ≈ 0,70-0,80 em mulheres em trabalho de parto)^(27)^.

Além desses instrumentos, foi aplicado questionário elaborado especificamente para este estudo, destinado a coletar informações sociodemográficas e clínicas da mãe e do bebê, utilizado para verificar critérios de elegibilidade, caracterizar a amostra e comparar os grupos na linha de base. O instrumento foi previamente avaliado por três especialistas em enfermagem pediátrica para adequação e clareza dos itens. As variáveis maternas incluíram idade (em anos), situação profissional (trabalhando fora de casa: sim/não), anos de escolaridade, renda familiar, número de moradores no domicílio e presença de doenças crônicas ou psicossomáticas (hipertensão, diabetes, doenças autoimunes, distúrbios do sono, ansiedade, depressão ou transtorno bipolar). Também foram coletadas informações sobre histórico de depressão pós-parto, tolerância ao choro do bebê e disposição para melhorar o sono da criança. Embora o foco deste estudo tenha sido a mãe, dados sobre o sono infantil, como número de despertares e tempo total de sono noturno, também foram obtidos por meio do BISQ.

### Análise estatística

Os dados foram exportados diretamente da plataforma *SurveyMonkey* ^®^ para planilhas em formato *Microsoft Excel* ^®^ e submetidos à dupla digitação independente, por dois pesquisadores, para minimizar erros. As inconsistências foram verificadas e corrigidas por meio de conferência cruzada. Antes das análises, os dados foram inspecionados para detecção de *outliers*, utilizando gráficos de caixa (*boxplots*) e escores z. Nenhum valor foi excluído por não apresentar impacto relevante na distribuição. As variáveis contínuas foram mantidas em formato numérico, e as categóricas foram codificadas com valores binários ou ordinais, conforme o tipo de análise requerida.

A normalidade das distribuições foi verificada pelo teste de Shapiro-Wilk. Para a caracterização da amostra, foram utilizadas estatísticas descritivas (médias, desvios padrão, frequências absolutas e frequências relativas). As comparações entre o GI e GC na linha de base e nos diferentes momentos de avaliação foram realizadas por meio do teste t para amostras independentes, para variáveis contínuas, e do teste qui-quadrado, para variáveis categóricas. Para analisar diferenças dentro de cada grupo ao longo do tempo (pré e pós-intervenção), utilizou-se o teste t pareado. A associação entre variáveis contínuas foi avaliada por meio da correlação de Pearson. O nível de significância adotado foi de 5% (p < 0,05). Todas as análises foram realizadas no *software* R (versão 4.2.1).

## RESULTADOS

A partir da divulgação do estudo nas redes sociais, 120 mulheres manifestaram interesse e preencheram os instrumentos de coleta entre março e junho de 2023. Dessas, 16 foram excluídas por não atenderem aos critérios de inclusão relacionados às características dos filhos. Assim, 104 díades mãe-bebê foram incluídas e randomizadas, sendo 52 alocadas no GI, e 52, no GC. A avaliação inicial (linha de base) foi realizada antes do início da intervenção. As participantes do GI foram reavaliadas aos 15 e 30 dias após a intervenção, enquanto as do GC foram reavaliadas aos 15 e 30 dias após a confirmação de participação.

A Figura 1 apresenta o fluxograma de recrutamento e acompanhamento das participantes em cada fase do estudo. As avaliações dos desfechos ocorreram em dois momentos: aos 15 e aos 30 dias após a intervenção para o GI; e após a confirmação de participação para o GC.

**Figura 1:** Fluxograma de recrutamento e alocação das participantes do estudo, Fortaleza, Ceará, Brasil, 2023

Na linha de base, verificou-se homogeneidade entre o GI e GC em relação s variáveis sociodemográficas da mãe (p>0,05), conforme apresentado na Tabela 1. Considerando as características sociodemográficas das mães de ambos os grupos, a média de idade foi de 31,2 (±3,6) anos; 59,6% tinham renda de três ou mais salários mínimos; 75% tinham entre 15 ou mais anos de estudo; 57,7% não trabalhavam fora de casa; e 75 % residiam na região Nordeste do Brasil.

**Tabela 1:** Características sociodemográficas e clínicas das mães segundo os grupos intervenção e controle na linha de base, Fortaleza, Ceará, Brasil, 2023 (N = 104)

Variáveis	Grupo intervenção (n=52)		Grupo controle (n=52)		Valor de *p*
	Média	Desvio padrão	Média	Desvio padrão	
Idade da mãe (em anos)	31,6	3,7	30,8	3,5	0,261^1^
Renda (salário R$1.302,00)^**^	3255,00	835	3330,00	791	0,639^1^
Escolaridade materna (anos)	14,9	1,89	15	1,74	0,872^1^
	n	(%)	n	(%)	
Trabalha fora de casa					
Sim	23	52,3	21	47,7	0,843^2^
Não	29	48,3	31	51,7	
Região do país					
Norte	4	7,7	1	1,9	0,362^3^
Nordeste	37	71,2	41	78,8	
Centro-Oeste	2	3,8	-	-	
Sudeste	8	14,4	8	15,4	
Sul	1	1,9	2	3,8	

1 teste t para amostras independentes;

2 qui-quadrado para homogeneidade;

3 qui-quadrado de Pearson com correção de Bonferroni;

** O salário mínimo no Brasil na época da coleta de dados era de R$ 1.302,00 (aproximadamente US$ 234, EUR 221 ou GBP 182).

A análise intragrupo, apresentada na Figura 2, evidenciou melhora progressiva e significativa na qualidade do sono e na ansiedade materna no GI ao longo dos 30 dias. O PSQI apresentou queda acentuada entre a linha de base e o 30º dia (p<0,001), enquanto que no GC, os escores permaneceram estáveis. Para a ansiedade-traço, observou-se redução significativa no GI (p<0,001), contrastando com o aumento progressivo no GC (p=0,012). A ansiedade-estado seguiu padrão semelhante, com melhora no GI (p=0,015) e piora no GC (p=0,011).

**Figura 2:** Comparação das médias dos escores de qualidade do sono (*Pittsburgh Sleep Quality Index*) e de ansiedade (Inventário de Ansiedade Traço-Estado) nos grupos intervenção e controle ao longo do tempo, Fortaleza, Ceará, Brasil, 2023

A Tabela 2 apresenta a comparação entre o GI e GC a partir dos escores totais do PSQI e IDATE. Também se apresenta a classificação conforme os pontos de corte dos instrumentos. Na linha de base, não houve diferenças significativas entre os grupos nos escores de sono (PSQI) e ansiedade (traço e estado), com ambos apresentando distúrbios de sono e ansiedade moderada. Após 15 dias, observou-se melhora significativa na qualidade do sono no GI (p<0,001), com escore médio indicando sono ruim e redução na ansiedade-estado (p=0,015). Aos 30 dias, o GI atingiu escore compatível com boa qualidade de sono (p<0,001), além de apresentar menores níveis de ansiedade-traço (p=0,009) e ansiedade-estado (p=0,007) em comparação ao GC, embora a classificação geral de ansiedade tenha permanecido como moderada.

**Tabela 2:** Comparação intergrupo segundo os escores do *Pittsburgh Sleep Quality Index* e Inventário de Ansiedade Traço-Estado na linha de base, com 15 e 30 dias após a intervenção, Fortaleza, Ceará, Brasil, 2023

	PSQI total	Classificação	Ansiedade-traço	Classificação	Ansiedade-estado	Classificação
**Momento/grupo**	**Média/DP**		**Média/DP**		**Média/DP**	
**Antes da intervenção**						
Grupo intervenção n=52	10,8±4,2	Presença de distúrbio de sono	46,5±6,5	Ansiedade moderada	43,6±6,3	Ansiedade moderada
Grupo comparação n=52	11,7±3,9	Presença de distúrbio de sono	46,9±6,3	Ansiedade moderada	44,2±6,4	Ansiedade moderada
Valor de p	0,292		0,774		0,610	
**15 dias após a intervenção**						
Grupo intervenção n=48	7,2±3,0	Sono ruim	45,6±6,7	Ansiedade moderada	42,9±4,3	Ansiedade moderada
Grupo comparação n=46	11,1±3,7	Presença de distúrbio de sono	47,7±6,6	Ansiedade moderada	45,8±5,7	Ansiedade moderada
Valor de p	**<0,001**		0,186		**0,015**	
**30 dias após a intervenção**						
Grupo intervenção n=45	4,8±2,0	Boa qualidade	44,9±6,3	Ansiedade moderada	42,8±3,1	Ansiedade moderada
Grupo comparação n=44	11,3±2,0	Presença de distúrbio de sono	48,5±6,4	Ansiedade moderada	48,8±5,1	Ansiedade moderada
Valor de p	**<0,001**		**0,009**		**0,007**	

* *teste t para amostras independentes; DP - desvio padrão; PSQI - Pittsburgh Sleep Quality Index.*

A Tabela 3 mostra correlações entre variáveis do sono infantil e os desfechos maternos no GI. Observou-se correlação negativa entre o tempo de sono noturno do bebê e os escores do PSQI materno aos 15 e 30 dias, indicando que quanto maior o sono do bebê, melhor a qualidade de sono da mãe. Também houve correlação positiva entre o número de despertares do bebê e os escores do PSQI materno, sugerindo pior qualidade de sono com mais interrupções. Além disso, o PSQI materno se correlacionou positivamente com os escores de ansiedade-traço nos dois momentos avaliados. Não foram encontradas correlações significativas com a ansiedade-estado.

**Tabela 3:** Correlação entre tempo de sono noturno, despertares de sono do bebê, qualidade de sono da mãe e ansiedade-traço-estado no grupo intervenção, Fortaleza, Ceará, Brasil, 2023

		Qualidade de sono mãe	Ansiedade-traço da mãe	Ansiedade-estado da mãe
**15 dias após a intervenção**				
Tempo de sono noturno do bebê	r^+^	-0,413	-0,252	-0,122
	p^*^	**0,004**	0,84	0,414
Despertares noturnos do bebê	r^+^	0,338	0,239	0,192
	p^*^	**0,019**	0,102	0,195
Qualidade de sono da mãe	r^+^	-	0,430	0,135
	p^*^		**0,003**	0,370
**30 dias após a intervenção**				
Tempo de sono noturno do bebê	r^+^	-0,422	-0,009	0,029
	p^*^	**0,004**	0,957	0,949
Despertares noturnos do bebê	r^+^	0,434	0,082	-0,37
	p^*^	**0,004**	0,599	0,812
Qualidade de sono da mãe	r^+^	-	0,492	-0,68
	p^*^		**0,001**	0,665

* *Correlação de Pearson; +coeficiente de correlação de Pearson.*

## DISCUSSÃO

Este estudo se destaca por sua abordagem inovadora ao integrar estratégias educativas *online* para melhorar a qualidade do sono e reduzir a ansiedade materna. A implementação de orientações práticas, como higiene do sono, técnicas de relaxamento e intervenções específicas para o sono e ansiedade materna, demonstra a viabilidade de intervenções remotas para mães. Esse modelo não apenas aborda questões imediatas relacionadas ao sono, mas também amplia horizontes, ao sugerir que outras temáticas, como saúde mental e bem-estar geral, possam ser tratadas em um formato semelhante.

A escolha por uma enfermeira especializada em sono foi um dos diferenciais da pesquisa. Esse suporte contínuo ajudou as mães do GI a implementar mudanças comportamentais com maior eficácia, resultando em uma redução significativa nas pontuações do PSQI e da ansiedade-traço. As mães receberam orientações detalhadas e personalizadas, o que fortaleceu a adesão às práticas propostas e contribuiu para resultados consistentes ao longo do estudo. A importância do acompanhamento especializado é amplamente corroborada pela literatura, que destaca que intervenções conduzidas por enfermeiras qualificadas aumentam a eficácia de programas educativos sobre sono^(28,29)^.

No início do estudo, ambas as mães do GI e do GC apresentavam PSQI > 10, indicando distúrbio de sono. Após 15 dias, as mães do GI já haviam reduzido suas pontuações para “sono ruim”, e, aos 30 dias, alcançaram “boa qualidade” (PSQI < 5). Em contrapartida, o GC manteve escores elevados, reforçando o impacto positivo da intervenção. Além disso, o GI apresentou reduções consistentes na ansiedade-traço, embora a ansiedade-estado tenha permanecido estável, possivelmente devido a influências externas não relacionadas diretamente às variáveis mensuradas no estudo.

Os encontros *online* permitiram um aprendizado dinâmico e acessível, ampliando o alcance do programa para mães em diferentes contextos geográficos do Brasil. Essa abordagem é especialmente relevante em um mundo cada vez mais digital, no qual barreiras geográficas podem ser superadas com o uso de tecnologias. Estudos prévios já apontaram que plataformas digitais são eficazes para promover saúde e bem-estar materno-infantil, principalmente quando combinadas com orientação profissional^(30,31)^.

Nas redes sociais, profissionais de saúde têm demonstrado capacidade de influenciar e engajar pessoas de distintos contextos socioculturais. Essa habilidade de comunicação e formação de vínculos pode ter contribuído para o alto engajamento das participantes e a baixa taxa de perdas amostrais ao longo do seguimento deste estudo. Estratégias semelhantes de promoção da saúde podem ser adaptadas ao contexto das Unidades Básicas de Saúde, onde enfermeiros e demais profissionais não precisam, necessariamente, utilizar suas redes sociais pessoais, mas podem mobilizar sua presença ativa, vínculo terapêutico e liderança comunitária para implementar ações educativas eficazes. Essa abordagem pode incluir temas ainda pouco explorados na atenção primária, como o sono materno-infantil, ampliando o cuidado integral e fortalecendo a promoção da saúde mental e do bem-estar familiar.

Adicionalmente, a relação entre o sono infantil e materno foi confirmada pelas correlações observadas. O tempo de sono noturno do bebê apresentou uma correlação negativa com o PSQI materno (ρ=-0,413 aos 15 dias; ρ=-0,422 aos 30 dias), enquanto os despertares noturnos tiveram correlação positiva (ρ=0,338 e ρ=0,434, respectivamente). Esses achados corroboram estudos que evidenciam a interdependência entre a qualidade do sono do bebê e da mãe, como relatado por Cai *et al*.^(9)^ e Sánchez-Garcia *et al*.^(32)^.

Outra contribuição relevante deste estudo foi o fortalecimento da autoeficácia materna. Embora esse construto não tenha sido diretamente mensurado, toda a intervenção educativa foi fundamentada em seus princípios, com o objetivo de promover a confiança das mães em sua capacidade de lidar com os desafios relacionados à melhoria da qualidade do sono e à redução da ansiedade. Esse foco na autoeficácia é um fator crítico para o sucesso de intervenções comportamentais, como também demonstrado por Schnatschmidt *et al*.^(16)^ e Carroll *et al*.^(13)^.

Estudos futuros devem explorar a sustentabilidade desses resultados a longo prazo, investigando se as melhorias na qualidade do sono e na redução da ansiedade são mantidas após a intervenção. Além disso, seria valioso aplicar essa abordagem em populações mais diversas, considerando variáveis culturais e socioeconômicas que possam influenciar os resultados. A integração dessa intervenção em programas de saúde pública também representa uma oportunidade promissora para ampliar seu impacto e beneficiar um número maior de famílias.

Deve-se destacar a relevância de integrar estratégias direcionadas ao sono e à ansiedade maternos, e ao sono infantil, nas políticas já existentes de saúde materno-infantil, como a Rede Cegonha e os programas de puericultura. A inclusão de ações específicas nesses protocolos, tais como educação sobre higiene do sono, identificação precoce de sinais de sofrimento emocional e suporte motivacional, pode contribuir para prevenir complicações decorrentes da privação de sono, cuja relação com desfechos adversos em populações maternas e infantis já foi amplamente demonstrada^(7,8)^. Este estudo reafirma que intervenções educativas *online*, conduzidas por profissionais especializados, podem promover mudanças significativas na qualidade de vida de mães e bebês. Ao abordar questões complexas, como sono e ansiedade de maneira acessível e baseada em evidências, esta pesquisa estabelece um modelo que pode ser replicado e expandido para outras áreas da saúde materno-infantil.

### Limitações do estudo

Este estudo apresenta limitações a serem consideradas. A coleta de dados foi realizada por meio de instrumentos autorreferidos, o que pode gerar vieses de memória e desejabilidade social, apesar do uso de escalas validadas. O registro da pesquisa na plataforma ReBEC ocorreu após o início da coleta devido a instabilidades técnicas, embora o protocolo tenha sido iniciado em novembro de 2022 e seguido conforme aprovado pelo Comitê de Ética em Pesquisa. A amostra, embora estatisticamente adequada, foi composta majoritariamente por mulheres do nordeste brasileiro com perfil socioeconômico semelhante, o que limita a generalização dos achados. Além disso, o período de seguimento de 30 dias impediu a avaliação da sustentabilidade dos efeitos a longo prazo, sugerindo a necessidade de estudos futuros com maior diversidade amostral e acompanhamento prolongado.

### Contribuições para a área da enfermagem, saúde ou políticas públicas

Este estudo oferece uma contribuição relevante para a enfermagem, ao demonstrar que intervenções educativas *online* podem ser eficazes na promoção da saúde do sono e na redução da ansiedade materna. Os resultados ampliam as possibilidades de atuação do enfermeiro, tanto ao incluir a temática do sono e da saúde mental materna em seu campo de cuidado quanto ao explorar novas formas de promover saúde por meio de tecnologias acessíveis.

Também auxilia na expansão de temáticas abordadas nas políticas públicas já existentes para a saúde materno-infantil no Brasil.

## CONCLUSÕES

Os resultados deste estudo confirmam a eficácia de intervenção educativa *online* conduzida por profissional especializado na melhora da qualidade do sono e redução da ansiedade materna. A abordagem focada na autoeficácia, aliada ao suporte técnico e emocional, demonstrou ser uma ferramenta valiosa para lidar com os desafios do sono materno. Além de promover benefícios imediatos, essa intervenção oferece um modelo replicável e escalável para outros contextos de saúde materno-infantil, ressaltando a importância da inclusão dessas estratégias em políticas públicas voltadas ao bem-estar das famílias. Estudos futuros devem explorar a sustentabilidade dos resultados e a ampliação dessa abordagem em populações mais diversas.

## Data Availability

Os dados de pesquisa só estão disponíveis mediante solicitação.
